# Synaptic scaling enables dynamically distinct short- and long-term memory formation

**DOI:** 10.1186/1471-2202-14-S1-P415

**Published:** 2013-07-08

**Authors:** Christian Tetzlaff, Christoph Kolodziejski, Marc Timme, Misha Tsodyks, Florentin Wörgötter

**Affiliations:** 1 Network Dynamics Group, Max Planck Institute for Dynamics and Self-Organization, Göttingen, Germany; 2 Institute for Physics III - Biophysics, Georg-August University, Göttingen, Germany; 3 Institute for Physics, Nonlinear Dynamics, Georg-August University, Göttingen, Germany; 4 Bernstein Center for Computational Neuroscience, Göttingen, Germany; 5 Weizmann Institute, Rehovot, Israel

## 

Memory formation in the nervous system relies on mechanisms acting on time scales from minutes, for long-term synaptic plasticity [1], to days, for memory consolidation [2]. During such processes, the neural network distinguishes synapses relevant for forming a long-term storage (LTS), which are consolidated, from synapses of short-term storage (STS), which fade. How time scale integration and synaptic differentiation is simultaneously achieved within one neural circuit remains unclear. We show in simulations and mean-field analyses that synaptic scaling [3] - a slow process usually associated with the maintenance of activity homeostasis - combined with the faster processes of synaptic plasticity simultaneously achieve both, thereby providing a natural separation of short- from long-term storage. A network intrinsic bifurcation enables this separation as this bifurcation induces different response properties of previously learned cell assemblies due to external memory reactivations. These reactivations could be associated with "sleep-like" activations as, for instance, sharp-wave ripples during slow-wave sleep [4,5]. Additionally, the interaction between plasticity and scaling provides an explanation for an established paradox where memory consolidation and destabilization critically depends on the exact order of learning and recall. This enables us to reproduce human-psychophysical results [6] on the apparently paradoxical effect of memory destabilization due to memory recall [7]. However, other experimentalists failed to reproduce this memory destabilization effect (e.g., [8]). This ambivalence can be explained by the here proposed bifurcation scenario as the initial conditions and exact timings of recall and learning determine the transition between consolidation and destabilization. Thus, the dynamics of our model yield the fact that memory - similar to the real systems - remains susceptible to perturbations and has to be repeatedly consolidated [2] which could happen during sleep [4,5]. To achieve a final stabilization of memory, systems consolidation, which also begins during sleep [4], performs a transition from a dynamic to a more static memory representation by transferring the information to the neocortex [2]. The processes suggested here are capable of repeatedly (re)consolidating LTS-synapses, while STS-candidates fade. This may thus essentially contribute to providing a stable substrate for systems consolidation.
